# Stromal Interferon Regulatory Factor 3 Can Antagonize Human Papillomavirus Replication by Supporting Epithelial-to-Mesenchymal Transition

**DOI:** 10.3390/v17050598

**Published:** 2025-04-23

**Authors:** Oluwamuyiwa T. Amusan, Rebecca Lopez, Elijah Burks, Jessica Trammel, Gaurav Raikhy, Hongyan Guo, Jason Bodily

**Affiliations:** Department of Microbiology and Immunology, Louisiana State University Health Sciences Center—Shreveport, 1501 Kings Highway, Shreveport, LA 71103, USA; oluwamuyiwa.amusan@lsuhs.edu (O.T.A.); jtramm4@lsu.edu (J.T.); gaurav.raikhy@louisiana.edu (G.R.); hongyan.guo@lsuhs.edu (H.G.)

**Keywords:** interferon regulatory factor 3 (IRF3), transforming growth factor beta 1 (TGFβ1), epithelial-to-mesenchymal transition (EMT), human papillomavirus type 16 (HPV16)

## Abstract

Epithelia contribute to the innate immune system through barrier formation and through signaling to immune cells. When the barrier is breached, epithelial cells undergo epithelial-to-mesenchymal transition (EMT) as part of the wound healing process. EMT is largely directed by signals from the stromal microenvironment, including transforming growth factor beta (TGFβ1), and antagonizes normal epithelial differentiation. How EMT and innate immunity may be connected molecularly has not been explored, although both processes are likely to occur simultaneously. Keratinocytes are the host cell type for human papillomaviruses (HPV), which can induce EMT in certain conditions but also depend on differentiation for their replication. We previously found that the innate immune factor interferon regulatory factor 3 (IRF3) inhibits epithelial differentiation and reduces the expression of HPV16 late genes. Here we report that IRF3 in the stroma compartment promotes an EMT-like pattern of gene expression in an HPV16-containing epithelium. The depletion of stromal IRF3 resulted in the downregulation of TGFβ1-related signaling in both the stroma and epithelium. IRF3 binds to the *TGFB1* promoter in human foreskin fibroblasts and is necessary for *TGFB1* mRNA production. Because an EMT-like state is unfavorable for differentiation-dependent HPV16, we observed that all EMT markers examined were reduced in the presence of episomal HPV16. Together, we show that stromal IRF3 can disrupt epithelial differentiation and act as an anti-HPV factor through the regulation of EMT, linking wound healing and immunity.

## 1. Introduction

Epithelia are critical tissues that form a barrier to protect the body from the outside world. Through various receptors present on their surface, epithelial cells can sense foreign agents or respond to environmental perturbations. As a result, epithelia are often the first line of defense against infection, injury, exposure to harmful agents, and other insults. Therefore, epithelia tend to be constantly renewed and maintained by stem cells, requiring a proper balance of proliferation, differentiation, repair, and responsiveness [1]. The stromal microenvironment is a community of cells, including fibroblasts, macrophages, endothelial cells, and immune cells [2], that play critical roles in maintaining tissue homeostasis through the regulation of tissue differentiation, wound healing, and epithelial-to-mesenchymal transition (EMT) [3]. During the wound healing process, stromal fibroblasts—the dominant stromal cells—in response to growth factors, including platelet-derived growth factor, transforming growth factor beta (TGFβ), and/or basic fibroblast growth factor (bFGF), migrate into the wound site and proliferate to establish the extracellular matrix, eventually sustaining de novo cellular growth, and mediating ECM remodeling and wound contraction [4,5,6,7]. Importantly, epithelial cells, in response to paracrine signals from the stroma, undergo EMT, a series of molecular events through which epithelial cells transdifferentiate towards a mesenchymal phenotype, detach from the basement membrane, and migrate into the wounded area to reconstitute the epithelial barrier [8]. EMT is characterized by increased motility, resistance to apoptosis, and invasiveness [9]. EMT has been implicated in embryogenesis [10], wound healing, fibrosis [11], inflammation [12], and cancer progression [13].

Although many extracellular factors can promote EMT, TGFβ is the most potent inducer [14,15]. TGFβ1 is the best-understood member of the TGFβ family [16]. Upon binding to several TGFβ receptors, TGFβ activates a signaling cascade that results in the eventual binding of SMAD transcription factors to TGFβ-responsive promoters to drive the transcription of EMT-related transcription factors (EMT-TFs), such as TWIST1/2, SNAI1/2, and ZEB1/2 [16,17]. These factors activate or suppress genes to promote EMT [17,18]. Of note, signaling events that culminate in EMT activation often overlap with the molecular mechanisms behind the regulation of cell survival, differentiation, adhesion, and motility [9,19]. Because stromal cells are the primary sources of TGFβ and other EMT-inducing molecules [15,20,21,22], the stromal microenvironment is an important tissue community that has developmental, physiological, and pathological implications. Much attention has been devoted to studying how stroma cells affect the microenvironment in the context of cancer or vice-versa [7,23,24,25,26]. There is a necessity for further research exploration in stroma-epithelium interaction, especially in non-tumor contexts.

Human papillomaviruses (HPVs) are non-enveloped, small DNA tumor viruses. HPV16 is among the high-risk HPVs; high-risk HPVs are responsible for essentially all cases of cervical cancer and an increasing share of oropharyngeal cancers globally [27]. Most cases of HPV infection do not result in cancer; under normal circumstances, the virus uses the stratified epithelium to produce more infectious virus particles. Following a microtrauma, the virus infects the rapidly dividing basal layer of a stratified squamous epithelium [28,29,30], where the viral genome is maintained in low copy numbers as an episome [31], regulated by viral E1 and E2 protein expression [32,33]. As differentiating cells move up from the basal layer to the suprabasal layers of the epithelium, they remain competent for DNA replication expression through the action of viral proteins E6 and E7. The initiation of replication is accompanied by the amplification of the HPV genome [34,35,36], and an increase in E1 and E2 expression [37]. Moreover, the differentiation-dependent E5 and E4 genes are expressed in the suprabasal and granular layers of the epithelium [38]. Eventually, capsid proteins L1 and L2 are synthesized in the highly differentiated granular layer, followed by the assembly of viral particles and release with the terminally differentiated corneocytes [38]. The HPV life cycle is thus heavily differentiation-dependent and therefore sensitive to changes in the epithelial phenotype of host keratinocytes.

In response to pathogens, the innate immune system is activated, and this is often characterized by the production of interferons. There are three types of interferon: type I, type II, and type III interferon [39]. Type I interferon family members include the well-studied IFNα and IFNβ, and the poorly defined IFNε, IFNτ, IFNκ, IFNω, IFNδ, and IFNζ [40]. Crucial to the production of type 1 interferon is the activation of interferon regulatory factor 3 (IRF3) [41,42]. Other notable IRF family members include IRF1, 5, 7, and 9 [43]. Mechanistically, upon the recognition of pathogen-associated molecular patterns by cognate pattern recognition receptors, IRF3 is phosphorylated downstream by TANK-binding kinase 1 (TBK1) or the inhibitor of NF-κB kinase ε/i (IKKε/i), eventually resulting in the dimerization and translocation of IRF3 to the nucleus, where it acts as a transcription factor to mediate the expression of type 1 interferon [42,44]. Consequently, IRF3 activation is thus vital for host defense against pathogens [41,45]. Of note, IRF3 is constitutively expressed in cells, and hence, besides its well-studied role in type 1 interferon induction and the production of inflammatory cytokines [46], there are other non-canonical functions of IRF3. IRF3 can decrease the activity of anti-apoptotic proteins, Mcl-1 and Bcl-xL [47,48], and increase proapoptotic Bax/Bak activity [49]. Similarly, other members of the IRF family have non-traditional cellular functions, such as in immune cell development and differentiation. IRF1, IRF2, IRF4, and IRF8 have been shown to be important in the development of dendritic cell subsets [43], while IRF5 and IRF4 drive macrophage polarization towards the M1 and M2 phenotype, respectively [50,51,52,53,54]. Put together, these reports suggest that other atypical functions of IRF3, such as in cellular differentiation, are possible.

**Figure 1:**
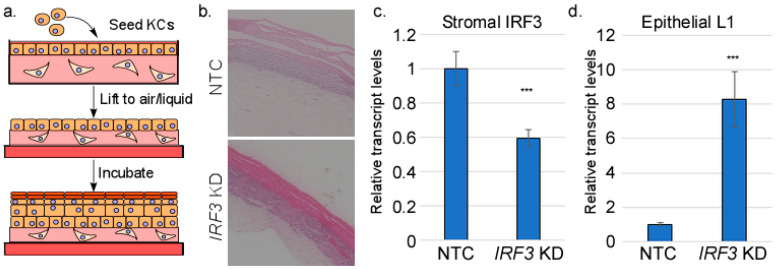
Stromal IRF3 suppresses HPV16 L1 transcript levels. (**a**) Preparation of organotypic (raft) cultures. HFFs are imbedded in a collagen matrix and keratinocytes are seeded on the surface. The construct is lifted onto a wire grid, exposed to the air on top, and fed by tissue culture medium from the bottom. During incubation, the cells proliferate, stratify, and differentiate. RNAs were isolated from either the stromal (fibroblast-containing) layer or the epithelial (keratinocyte-containing) layer of raft cultures made from HFFs in which *IRF3* was knocked down or HFFs containing non-target control shRNA (NTC). (**b**) Representative H&E images of NTC and *IRF3* KD raft cultures. RNAs were subjected to RT-qPCR analysis to measure levels of (**c**) stromal *IRF3* or (**d**) epithelial *L1* RNAs. N = 3. *** = *p* < 0.001.

We and others have previously used organotypic raft cultures to investigate the HPV life cycle, including potential crosstalk between HPV-containing epithelium and the stroma (Figure 1a) [55,56,57,58,59,60]. The organotypic raft culture is an in vitro 3D culture system with skin-like architectural properties. It is composed of two major cell types: fibroblasts and keratinocytes. The fibroblasts—the dominant stromal cells—are mixed with a solid matrix (such as collagen) to form the dermal equivalent. Keratinocytes are then seeded on the dermal plug and allowed to attach. The construct is then lifted onto an air-liquid interface, allowing for proper keratinocyte differentiation into an epithelium. This system supports the complete in vitro life cycle of HPV which is heavily differentiation-dependent [61]. We have previously investigated epithelial–stromal interaction using this model, showing that the presence of HPV16 in the epithelium can affect several stromal pathways, with the IFN-regulated genes being suppressed in the stroma [55]. Surprisingly, we found that disruption of IRF3 in the stroma did not impact the expression of IFNs themselves or interferon-stimulated genes to a significant extent. Rather, knockdown of IRF3 promoted epithelial differentiation, leading to higher levels of differentiation-dependent HPV16 L1 expression [55]. In this study, we followed up on this atypical activity of IRF3 and showed that IRF3 in the stroma can promote an EMT-like pattern of gene expression in the epithelium, suggesting a possible molecular connection between innate immune responses and wound healing.

## 2. Materials and Methods

### 2.1. Cell Lines and Cell Culture

Human foreskin fibroblasts (HFFs) and human foreskin keratinocytes (HFKs) were derived from discarded and deidentified neonatal foreskins, as previously described [55]. HFFs were cultured and maintained in Dulbecco’s modified Eagle’s medium (DMEM), supplemented with 10% bovine growth serum (BGS). HFKs were cultivated in E medium and supplemented with 5% fetal bovine serum (FBS) in the presence of mitomycin-treated HFFs [55]. To obtain HFKs stably harboring the HPV16 genome, HFKs were transfected with a plasmid containing HPV16, as previously described [62]. The episomal status of the HPV16 genomes was confirmed by Southern Blotting or by inducing the differentiation of the HPV16-containing HFKs in a semi-solid methylcellulose-containing medium for 24 h, with the differentiation-dependent viral transcripts measured by RT-qPCR [63].

### 2.2. Organotypic Raft Culture

To generate organotypic rafts, low passage-number HFKs or HFKs harboring HPV16 (with confirmed episomal status) were used as previously described [64]. For all the rafts used in this study, low-passage-number HFFs (rather than murine J2 cells) were used to form the dermal equivalent. At least three different human foreskin fibroblast (HFF) lines and three human foreskin keratinocyte (HFK) donor backgrounds were used for all the raft experiments.

### 2.3. Immunofluorescence

Immunofluorescence staining of cells in monolayer or fixed organotypic raft tissues was performed as described in [56] using antibodies described in Supplementary Table S1. Confocal images of monolayer immunofluorescence staining were captured on a Leica TCS SP5 Confocal Microscope (Leica, Deerfield, IL, USA) at 100× magnification with oil immersion. Image analysis was performed using IMARIS (version 9.9.1). All images were subjected to the same image processing and thresholding. Images of stained raft tissues were captured using a Keyence Fluorescence Microscope BZ-X800 (Keyence, Itasca, IL, USA) with a 40× lens, and raw image quantification was performed using ImageJ software (version 1.53e).

### 2.4. RNA-Seq

Total RNAs were isolated and subjected to RNA-seq analysis. Total RNA integrity was assessed on an TapeStation 2200 (Agilent, Santa Clara, CA, USA) using an RNA ScreenTape assay (Agilent). Libraries were prepared using Illumina’s TruSeq Stranded mRNA LS kit (Illumina, San Diego, CA, USA) according to the manufacturer’s protocol. Libraries were analyzed on a TapeStation 2200 D1000 assay (Agilent) to determine average size and were quantitated by qPCR using the NEBNext Library Quant Kit (New England Biolabs, Ipswich, MA, USA). The libraries were combined into one of two pools: the stromal or epithelial pool. Libraries were normalized to 4 nM, pooled, denatured, and diluted to approximately 1.8 pM. A 1% library of 1.8 pM PhiX was spiked in as an internal control. The library pools were sequenced on an NextSeq 550 (Illimina), with a read length of 75 base pairs, on two cartridges. Base calling and quality scoring were performed with Illumina Real Time Analysis software (RTA, version 2.4.11). Sample reads from fastq files were consolidated. Reads were aligned to a concatenated human genome (ChrGRCh38p12v30) and HPV 16 genome (HPV16-NC_0015263) with STAR 2.5.31. Transcripts were quantified with RSEM 1.3.0. Lists of differentially expressed transcripts, at an FDR of 0.05, were generated with EBSEQ 1.2.0.

### 2.5. RNA Extraction and RT-qPCR

For monolayer cells, RNA-STAT 60 (TelTest, Inc., Friendswood, TX, USA) was used to isolate the total RNA according to the manufacturer’s directions. For organotypic raft cultures, the epithelial layer was manually separated from the stromal layer. Total RNA was isolated from each component by the Feist-Weiller Cancer Center Tissue and Serum Repository Genomic Isolation core lab on a Qiagen Qiacube (Germantown, MD, USA), as described previously [55]. Following this, the RNAs were converted to cDNAs using qScript (Quanta, Beverly, MA, USA), as described previously [65]. To perform the quantitative PCR, we used PerfeCTa SYBR Green SuperMix ROX (Quanta) on an Applied Biosystems StepOne Plus real-time PCR machine using the primers shown in Supplementary Table S2. Integrated DNA Technologies (IDT, Coralville, IA, USA) synthesized all the primers. We measured fold changes using the ΔΔ CT method, and cyclophilin A (PPIA) was used as a housekeeping control.

### 2.6. Immunoblotting

Cell Lysis Buffer (Cell Signaling, Danvers, MA, USA) was used to isolate the total protein. Using Bradford’s assay (Bio-Rad, Hercules, CA, USA), we measured the protein concentration, and subsequently used about one hundred micrograms of total protein for gel electrophoresis (using sodium dodecyl sulfate-polyacrylamide gel). Protein was transferred to a pre-wet polyvinylidene difluoride (PVDF) membrane at 100 V for 1 h; followed by a 1-hr incubation with LI-COR Intercept Blocking Buffer. After at least three 5-min washes, the membrane was incubated with antibodies listed in Supplementary Table S1, diluted in LI-COR Intercept Blocking Buffer (with 0.1% Tween) overnight, washed (3×, 5 min intervals) and incubated with near-infrared (IR) secondary antibodies (LI-COR, diluted in LI-COR Intercept Blocking Buffer with 0.1% Tween) for 1 hr. The membranes were then washed and scanned with a LI-COR Odyssey system. Image Studio 5.2 was used for quantification, and the expression levels of the protein were normalized to GAPDH (glyceraldehyde-3-phosphate dehydrogenase) levels.

### 2.7. Chromatin Immunoprecipitation

Chromatin immunoprecipitation (ChIP) was performed as described previously [66]. Briefly, HFFs were trypsinized and incubated with formaldehyde (1% final) to crosslink the chromatin. Following quenching with glycine, cells were washed and resuspended in Cell Lysis Buffer (Cell Signaling) to a final density of 10 million cells/mL. Chromatin was treated briefly with micrococcal nuclease, cleared by centrifugation, diluted, and subjected to 1 h preclearing with IgG-coupled Protein G Dynabeads. Anti-IRF3-coupled Dynabead complexes were added and incubated overnight, followed by a series of washes, reversal of crosslinking, and purification of DNA fragments. Immunoprecipitated DNA fragments were subjected to qPCR using primers listed in Supplementary Table S2. Values were normalized to the IgG background control.

### 2.8. siRNA Transfection

*IRF3* was targeted with ON-TARGETplus SMARTpool L006875000005 and the negative control was D-001810-10-20 (both from Dharmacon, Lafayette, LA, USA). The DharmaFECT siRNA protocol was followed. Briefly, cell lines were seeded into a 6-well plate at 250,000 cells/well and incubated with DMEM + 10% BGS. Twenty-four hours post-seeding, the siRNA was diluted to 35 nM in Opti-mem (Gibco, Grand Island, NY, USA, #11058-021) using DharmaFECT1 (Dharmacon, Lafayette, USA, T-2001-02) at a concentration of 50 μL/mL and added to cells incubated with DMEM + 10% BGS, according to manufacturer’s protocol. Cells were harvested for RNA or protein 72 h post-transfection.

### 2.9. shRNA Lentivirus Transduction

3 million HFFs were seeded in 10 cm dishes. Following overnight incubation, 1 mL *IRF3*-Sigma Aldrich (St. Louis, MO, USA)-TRCN0000005921, TRCN0000005923, and TRCN0000005919) or *TGFB1*-(Sigma Aldrich-TRCN0000003316) targeting lentiviral suspensions (supplemented with 5 µg/mL polybrene) were added to the HFFs. The cells were then incubated overnight and then supplied with fresh media. After 24–48 h, the cells were supplied with fresh DMEM+10% BGS, supplemented with puromycin (2 µg/mL) for selection. Selected surviving cells were then expanded for downstream experiments.

### 2.10. Statistics

All experiments were conducted a minimum of three times, usually more, using cells from at least three independent donor backgrounds to control for donor-to-donor variability. The significance of the quantifications was calculated using Welch’s unequal variances *t*-test. NS = not significant; * = *p* < 0.05; ** = *p* < 0.01; *** = *p* < 0.001.

## 3. Results

We have previously shown that stromal IRF3 depletion resulted in elevated levels of both HPV16 late gene transcripts and transcripts of cellular differentiation markers, suggesting a possible suppression of the late stages of keratinocyte differentiation by IRF3 [55]. In the current work, we sought to delineate the mechanisms behind this suppression and identify the major players. Organotypic cultures were created using control human fibroblasts in the stroma, or fibroblasts in which IRF3 transcripts were reduced using shRNAs. Human foreskin keratinocytes immortalized by episomally replicating HPV16 comprised the epithelium (Figure 1a). Organotypic rafts were grown for 14 days, which was sufficient for the formation of stratified epithelium (Figure 1b). We obtained the total RNA from the epithelium and measured the late viral transcripts by RT-qPCR. Consistent with our previous finding, we observed a robust increase in HPV16 highly differentiation-dependent L1 transcripts upon stromal IRF3 knockdown (Figure 1d), confirming the observation that the knockdown of stromal IRF3 increases late keratinocyte differentiation and promotes the expression of late viral genes.

**Figure 2:**
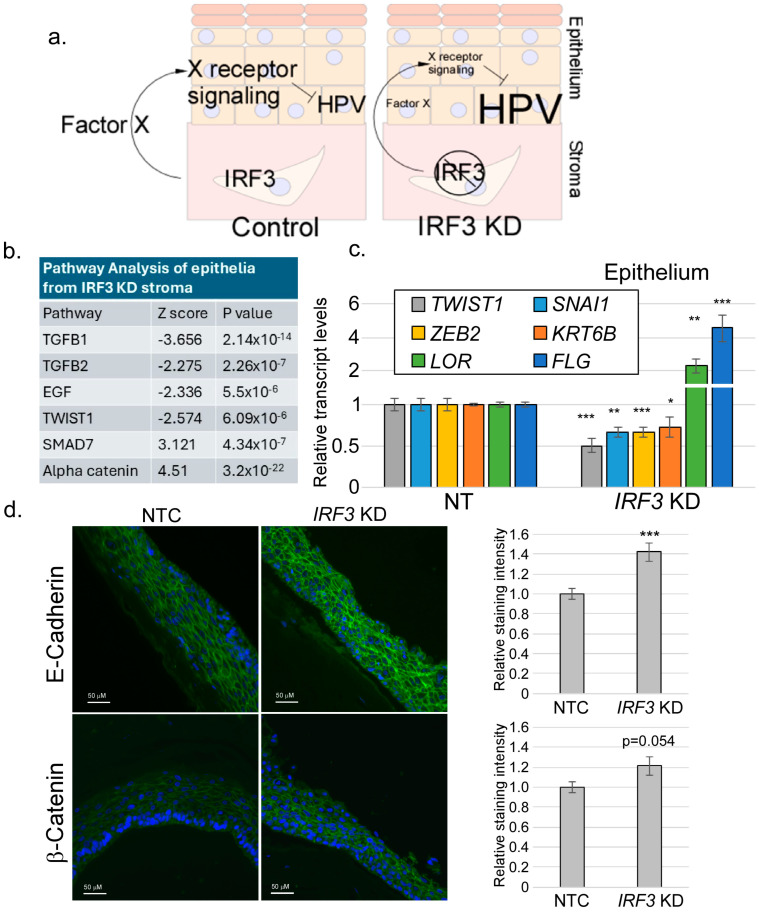
Stromal IRF3 promotes an EMT-like gene expression pattern in the epithelium. (**a**) Hypothesis: IRF3 supports expression of a paracrine factor (X) whose downstream signaling suppresses HPV late gene expression. Knockdown of IRF3 would reduce levels of X, resulting in increased HPV late gene expression. Total RNA-seq was performed on RNAs from both the epithelial and stromal compartments of rafts with or without *IRF3* knockdown in the stromal fibroblasts (N = 3 each). Keratinocytes contained episomal HPV16. (**b**) Pathway analysis from epithelial RNAs showing a subset of pathways regulated by stromal IRF3. (**c**) RT-qPCR analysis of selected RNAs from the epithelial layer. N = 6. (**d**) Raft cultures were processed, imbedded, sectioned, and stained using antibodies against E-cadherin (**top**, green) or β-catenin (**bottom**, green). Intensity of staining was quantified (**right**). N = 4. NS = not significant; * = *p* < 0.05; ** = *p* < 0.01; *** = *p* < 0.001.

We sought to determine the mechanism by which stromal IRF3 suppresses differentiation. Because the stroma and the epithelium are separated by a basement membrane, we assumed that the mechanism of IRF3-induced suppression of differentiation was through a paracrine factor regulated by IRF3 (Figure 2a). We became highly interested in identifying this factor since it could have the potential to disrupt HPV replication. Organotypic rafts were created as described above. Following 14 days of culture, total RNAs were isolated from both the epithelial and stromal compartments and RNA-seq analysis was performed (Supplementary Table S3). We reasoned that a paracrine signal emanating from the stroma would result in changes in signaling in the epithelium. Candidate pathways were identified by performing pathway analysis on the epithelial transcript sequences. Then, agonists or antagonists of those pathways were identified in the stromal sequences.

Because of the role of IRF3 in driving the production of IFN, we initially hypothesized that the stromal signal suppressing differentiation would be one or more IFNs. To our surprise, we found that the IFN expression in both the stroma and epithelium was extremely low and did not increase or decrease significantly by the knockdown of IRF3 (Supplementary Table S4). This finding suggested that stromal IRF3 promotes the expression of a non-IFN factor that regulates epithelial differentiation.

Pathway analysis data revealed that, upon stromal depletion of IRF3, components of the TGFβ1 signaling and related pathways (such as GDF and BMP) were significantly downregulated in the epithelium (Figure 2b). The SMAD7 pathway, which antagonizes TGFβ1, was upregulated. The EMT transcription factor TWIST1, part of a pathway regulated by TGFβ1, was also downregulated. Since the TGFβ1 signaling axis has been widely reported to be the inducer of the EMT process, including *TWIST1*, we proceeded to examine EMT-related transcript levels in the epithelium upon stromal IRF3 depletion. We observed a significant decrease in the transcript levels of EMT factors such as *TWIST1*, *ZEB2*, *KRT6B*, and *SNAI1* in the epithelia of *IRF3*-knockdown rafts (Figure 2c); at the same time, there was a noticeable increase in the levels of the late differentiation transcripts *FLG* and *LOR*. E-cadherin and Beta-Catenin staining intensity within the epithelium (Figure 2d), consistent with a shift toward a more epithelial phenotype in stromal IRF3-KD rafts.

**Figure 3:**
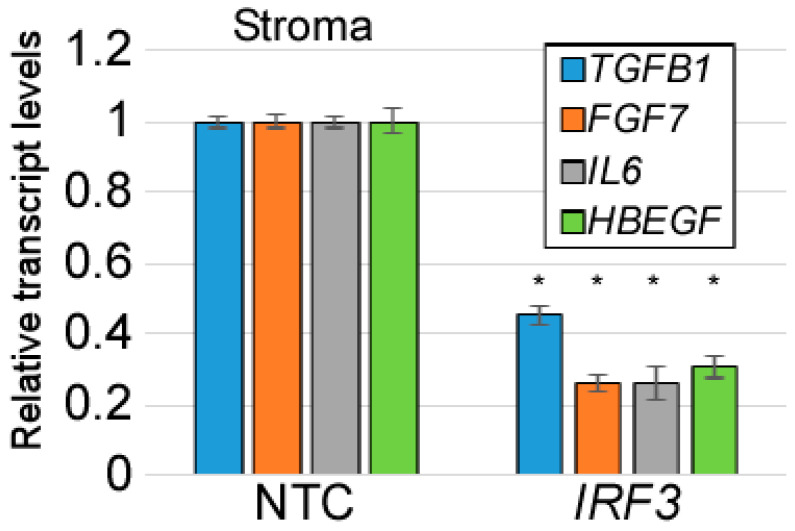
Stromal IRF3 promotes expression of cytokines and growth factors in the stroma. RNAs were isolated from the stromal layer of rafts containing HFFs with IRF3 knocked down (or controls) and subjected to RT-qPCR analysis. N = 4. * = *p* < 0.05.

Turning to gene expression in the stromal fibroblasts in cultures with reduced *IRF3*, we noticed a significant reduction in the mRNA levels of EMT inducers, including *TGFB1* (Figure 3). These findings together suggest that IRF3 promotes an EMT or EMT-like condition by promoting the expression of TGFβ1 or another EMT inducer. Notably, the EMT process is antagonistic to epithelial differentiation. Hence, our data suggest that stromal IRF3-mediated suppression of HPV16 late gene expression is through promoting EMT at the expense of differentiation.

**Figure 4:**
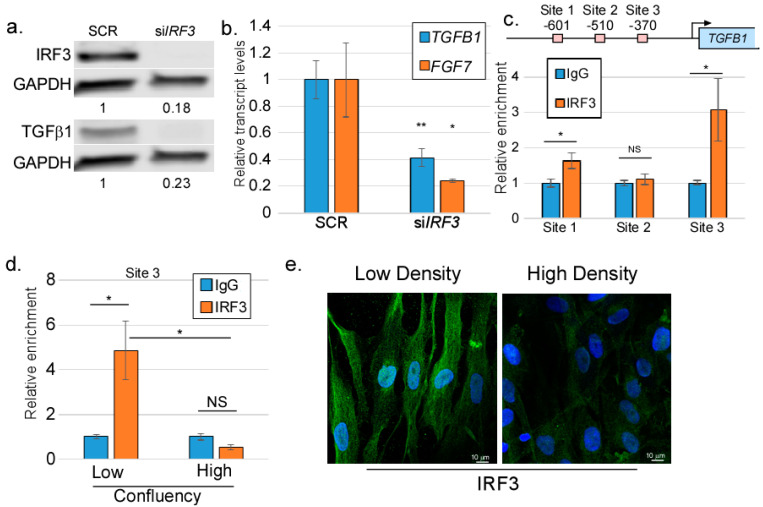
IRF3 is necessary for TGFβ1 expression in isolated fibroblasts. HFFs were transfected with either *IRF3*-specific siRNAs or scrambled controls. (**a**) Total protein was harvested from transfected cells and immunoblotted for IRF3 (**top**) and TGFβ1 (**bottom**) with GAPDH as a loading control. Mean band intensities are indicated below the blots (N = 5). (**b**) Total RNAs from transfected cells were subjected to RT-qPCR analysis for *TGFB1* and *FGF7* transcripts (N = 6). (**c**) (**Top**) putative IRF3 binding sites in the *TGFB1* promoter. (**Bottom**) Chromatin was prepared from HFFs and subjected to chromatin immunoprecipitation, followed by PCR for each putative IRF3 binding site. (**d**) Chromatin was prepared from untreated HFFs grown at either high or low density and subjected to chromatin immunoprecipitation using antibodies targeting IRF3. PCR was performed on DNA isolated from the immunoprecipitates using primers targeting Site 3 (**top**). N = 3. (**e**) HFFs were grown at either high or low density, fixed, and stained with antibodies against IRF3 (green), with DAPI counterstain (blue). N = 3. NS = not significant; * = *p* < 0.05; ** = *p* < 0.01.

As there was no published data suggesting that IRF3 could regulate *TGFB1*, we focused our efforts on determining the relationship between IRF3 and *TGFB1* expression. First, we wondered if IRF3 regulates the expression of the *TGFB1* gene in isolated primary human foreskin fibroblasts (HFFs), or whether the presence of an epithelium is required. We used siRNA to deplete *IRF3* in HFFs and measured the transcript and protein levels of TGFβ1. Strikingly, our data revealed that IRF3 knockdown in HFFs corresponded with a decrease in *TGFB1* transcript and TGFβ1 protein levels, suggesting that IRF3 is required for the expression of TGFβ1 in these cells (Figure 4a). IRF3 was also important to support the expression of other EMT-inducing factors, such as *FGF7* (Figure 4b). The *TGFB1* promoter region contains three potential IRF3 binding sites (Figure 4c) and so we used chromatin immunoprecipitation to determine if IRF3 can bind to any of these sites. Our data revealed that IRF3 binds to two out of three of these sites, with the highest IRF3 enrichment found on binding Site 3 (Figure 4c). Interestingly, the ability of IRF3 to bind to the *TGFB1* promoter was dependent on the cellular density, with binding being more evident in cells that were at low confluency (Figure 4d). Using immunofluorescence staining, we found that HFFs at low confluency harbored higher levels of IRF3, with a significant proportion of the IRF3 being found in the nucleus; at high confluency, however, the levels of IRF3 were lower, and very little was nuclear (Figure 4e). Taking these data together, we conclude that IRF3 can bind to the *TGFB1* promoter and is required for TGFβ1 production, suggesting that IRF3 can directly regulate the expression of *TGFB1* transcription.

**Figure 5:**
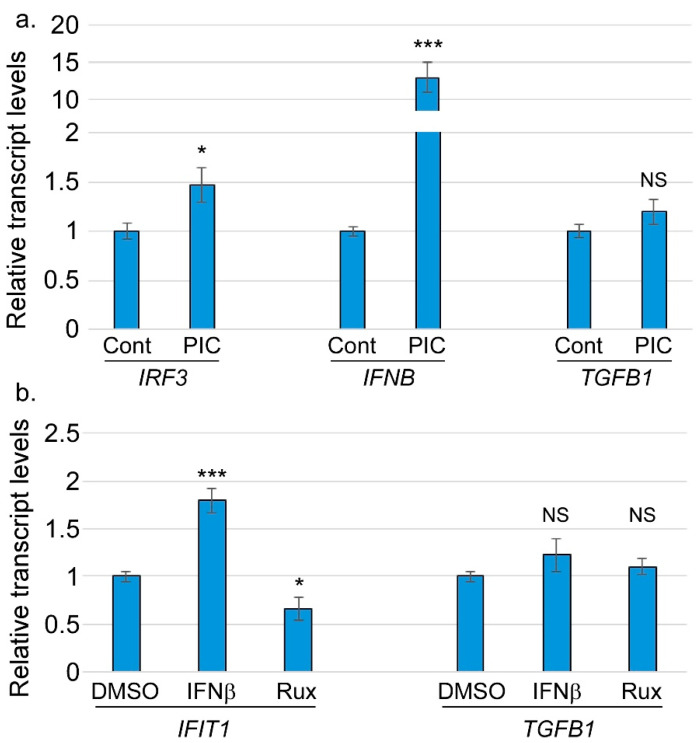
*TGFB1* gene expression does not respond to the canonical IRF3 pathway. HFFs were treated with (**a**) poly I:C (PIC), (**b**) IFNβ (25 U/mL), or ruxolitinib (10 µM) for 24 h. Total RNAs were subjected to RT-qPCR analysis using the indicated primers. N = 3. NS = not significant; * = *p* < 0.05; *** = *p* < 0.001.

Notably, these experiments were not conducted under conditions that stimulate IRF3 through the classic pattern recognition receptor pathway, leading us to wonder about the relationship between TGFβ1 regulation and the other canonical functions of IRF3. If *TGFB1* is activated by IRF3, we wondered whether *TGFB1* might act as part of the interferon response. IRF3 is activated upon stimulation with pattern recognition receptor agonists, so we tested whether the exogenous stimulation of IRF3 would increase *TGFB1* transcript levels. When HFFs were treated with a previously reported IRF3 agonist polyinosinic-polycytidylic acid (poly(I:C)), we observed that *IRF3* and *IFNB* levels both increased significantly but *TGFB1* levels did not (Figure 5a). We interpreted this to mean that *TGFB1* does not respond to the stimulation of IRF3 through the classic pattern recognition receptor pathway. To test if the IRF3-dependent expression of TGFβ1 is downstream of an interferon response, or whether TGFβ1 itself is an interferon-stimulated gene (ISG), we treated HFFs with interferon β (IFNβ) and measured the transcript levels of *TGFB1* and *IFIT1* (a typical ISG). Our findings revealed significantly increased transcript levels of *IFIT1*, while *TGFB1* levels remain unchanged (Figure 5b). The JAK/STAT pathway acts downstream of the type I interferon receptor to drive the expression of ISGs. HFFs treated with ruxolitinib (a JAK/STAT inhibitor) had significantly reduced *TGFB1* and *IFIT1* transcript levels, while *TGFB1* levels again were unchanged. Taken together, these findings show that the IRF3-dependent regulation of *TGFB1* is not through IFN and JAK-STAT signaling. 

**Figure 6:**
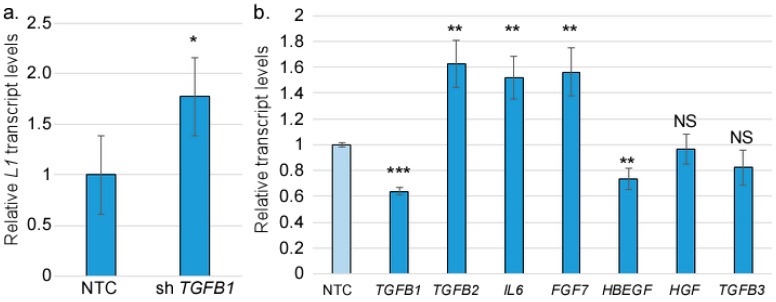
TGFβ1 contributes to a network of growth factors in the stroma. RNAs were harvested from raft cultures containing HFFs with *TGFB1* knocked down (or controls). (**a**) RNAs from the epithelium were subjected to RT-qPCR analysis to measure the levels of *L1*. N = 6. (**b**) Levels of the indicated transcripts in the stroma of *TGFB1* KD or NTC rafts were measured by RT-qPCR analysis. N = 8. NS = not significant; * = *p* < 0.05; ** = *p* < 0.01; *** = *p* < 0.001.

So far, our findings reveal that IRF3 suppresses the late stages of the HPV16 life cycle, and that IFR3 is required for the basal TGFβ1 levels in fibroblasts. The HPV16 life cycle is heavily dependent on differentiation, and as expected, an induction of the EMT process correlated with an inhibition of differentiation, as reflected in the increased levels of L1 transcripts upon stromal IRF3 depletion. We hypothesized that a reduction in TGFβ1 levels should mirror IRF3 depletion. To test this hypothesis, we used lentiviral shRNA to knockdown *TGFB1* in human foreskin fibroblasts. The *TGFB1*-depleted HFFs or controls were then grown with HPV16-containing human foreskin keratinocytes in an organotypic raft model. After 14 days, the epithelial RNAs were harvested and subjected to RT-qPCR. Consistent with stromal *IRF3* depletion, the reduction in stromal *TGFB1* levels resulted in an upregulation of HPV16 late viral transcript, *L1*, although to a lesser degree than the knockdown of IRF3 (Figure 6a). However, we did not observe a significant increase in *FLG* levels in *TGFB1* KD raft cultures. We considered whether there was a possible compensatory effect from other EMT inducers resulting from stromal TGFβ1 depletion. We found a significant increase in the transcript levels of *TGFB2*, *IL6*, and *FGF7* upon *TGFB1* depletion (Figure 6b). Like *TGFB1*, these transcripts were also dependent on IRF3 levels (Figure 3). We concluded that IRF3 is a critical factor driving a network of EMT-inducing growth factors in the stroma.

**Figure 7:**
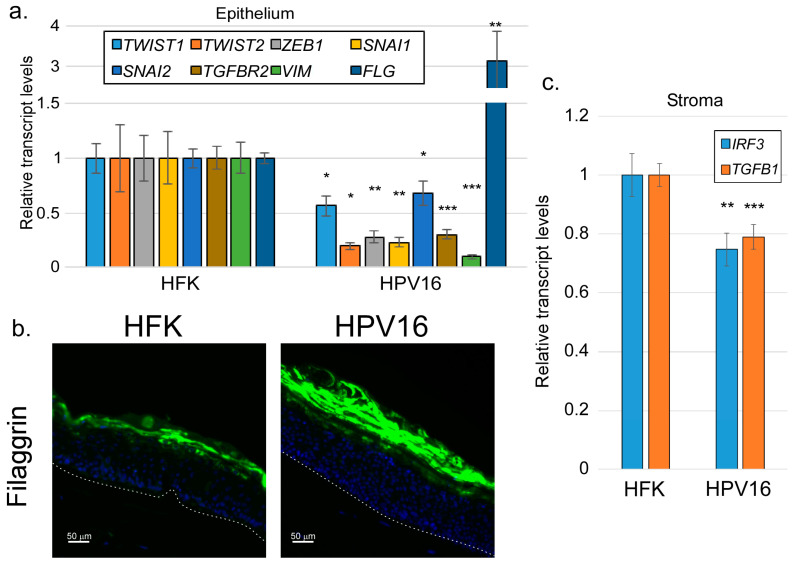
HPV16 suppresses EMT-related factors in the epithelium and *IRF3*/*TGFB1* in the stroma. Raft cultures containing either uninfected HFKs or HPV16-containing keratinocytes were harvested for RNA isolation or sectioning. (**a**) RNAs from the epithelial compartment were subjected to RT-qPCR analysis using the indicted primers. N = 4. (**b**) Fixed sections were stained using antibodies specific for filaggrin (green). The dotted line represents the basement membrane. (**c**) RNAs from the stromal fibroblasts were subjected to RT-qPCR using the indicated primers. N = 9. NS = not significant; * = *p* < 0.05; ** = *p* < 0.01; *** = *p* < 0.001.

As carcinogenic agents, HPV oncogenes have been repeatedly shown to induce EMT-like effects in infected cells [67,68,69]. However, the HPV life cycle depends critically on cellular differentiation. Our findings so far have suggested that signals promoting EMT in the epithelium may have an antiviral effect, suppressing the late stages of the viral life cycle. If so, we might expect that productively replicating HPV would suppress rather than promote EMT. We grew organotypic raft cultures using either uninfected HFKs or HFKs maintaining episomal HPV16 genomes. Following 14 days of culture, we examined the levels of various EMT-related factors in the epithelium using RT-qPCR (Figure 7a). We found that all EMT markers examined were reduced by the presence of episomal HPV16, including *TWIST1/2*, *SNAI1/2*, *ZEB1*, and *VIM*. As shown previously [70], HPV16 also reduced the transcript levels of *TGFBR2*, one of the main components of the TGFβ receptor. Consistent with our previous work [56], HPV16 rafts expressed higher levels of *FLG* transcripts and filaggrin protein (Figure 7a,b), consistent with the idea that episomal HPV16 shifts the balance away from EMT and toward differentiation of the epithelium. Finally, we found that the levels of *IRF3* and *TGFB1* transcripts in the stroma of HPV16-containig rafts were modestly but significantly lower than the levels found in rafts containing uninfected HFKs (Figure 7c), indicating that HPV16 in the epithelium suppresses these factors in the adjacent stroma.

## 4. Discussion

Keratinocytes engage a two-way communication with the stromal cells in the underlying dermis, often resulting in physiological and/or pathological implications [71]. Most studies have examined this stroma–epithelium interaction in tumor contexts, with stroma cells reported to worsen cancer progression/metastasis [7,23,24,26]. However, stromal–epithelial interactions are also critical in normal physiological processes, such as wound healing [71,72,73]. Productive HPV infections are not cancers, so it cannot be assumed that the interactions between HPV-infected stroma are the same as those found in tumor contexts. Previously, we reported that the depletion of IRF3 in stromal fibroblasts resulted in a corresponding increase in HPV16 late gene transcripts and cellular differentiation markers, suggesting a possible paracrine regulation of keratinocytes by IRF3 in stromal fibroblasts [55]. In the present study, using organotypic raft cultures with depleted or normal stromal IRF3, we confirmed that IRF3 suppresses late viral transcripts of HPV16 (Figure 1), suggesting a possible IRF3-mediated restriction of the viral life cycle within the host. This finding is consistent with the widely reported roles of IRF3 in antiviral defense [41,45,74,75], but our findings suggest a new potential mechanism for IRF3-mediated antiviral defense, namely the promotion of EMT. Our RNA-seq analyses surprisingly revealed that IRF3 is important for supporting TGFβ-related signaling pathways. Consistently, factors or inducers of EMT, a process regulated by TGFβ signaling, were also significantly downregulated upon loss of IRF3 both in the epithelium and in the stroma, suggesting that IRF3 can promote the EMT process, a condition unfavorable for epithelial differentiation, and consequently, HPV16 late gene expression. Mirroring our observations, Ni et al. [76] reported an in vitro study in which the depletion of IRF3 decreased the expression of important wound healing factors, α-SMA and Colla1, in TGFβ1-induced LX-2 cells. In contrast, IRF3 was also reported to inhibit TGFβ signaling by competitively inhibiting SMAD3, a transcription factor critical for TGFβ signaling, resulting in the repression of TGFβ signaling-mediated processes such as EMT, and regulatory T cell differentiation [77]. While Ni et al. [76] examined the basal activity of IRF3 in immortalized liver cells, Xu et al. [77] applied viral RLR agonists or mimics to activate IRF3 and then assessed the downstream effect on TGFβ-mediated signaling activities. The discrepancy in the role of IRF3 may therefore be due to the status of IRF3 activation in cell-specific contexts.

Using siRNA and ChIP experiments, we found that IRF3 can directly bind to and drive the expression of the *TGFB1* gene. Of note, apart from binding to ISRE promoter elements [78], IRF3 has been reported to bind ISRE-like elements and activate the RANTES promoter [79], or bind to IRF3-binding sites within the SCD1 promoter to repress *Scd1* transcription and prevent hepatic steatosis [80]. These findings suggest a transcriptional activity of IRF3 that extends beyond interferon genes alone. We found that *TGFB1* regulation by IRF3 does not require, nor does it respond to classical IRF3 stimulation. Whether IRF3-mediated regulation of *TGFB1* requires IRF3 activation and phosphorylation remains to be understood. Our findings suggest that basal IRF3 activity positively regulates the TGFβ signaling axis, consistent with a previously observed IRF3-mediated effect on TGFβ signaling, where IRF3 drives the expression of TGFβ1-induced factors such as Col1a1 [76]. We also observed an effect of cell density on IRF3 localization to the nucleus of cultured HFFs. We do not yet understand the significance of this finding.

TGFβ1 is the most potent EMT inducer [14]. While we observed a significant increase in L1 transcripts upon knockdown of stromal TGFβ1 [56], the transcript levels of all the differentiation markers checked were either unchanged or slightly increased. These findings suggest either that TGFβ1 itself is not the critical factor regulating the EMT downstream of stromal IRF3 or that *TGFB1* depletion resulted in a compensatory effect that then caused an increase in other TGFβ receptor ligands or other pathways. In agreement, we found that other EMT ligands, such as TGFβ2, IL6, and FGF7 [81,82], were increased upon TGFβ1 depletion. Understanding the role of this network of signaling factors will require additional research.

Finally, we found that HPV16 infection in the epithelium suppressed the levels of a variety of EMT-related transcripts. Because EMT is often associated with tumorigenesis [13], and overexpressed HPV oncoproteins can promote EMT [67,68,69], it may be surprising that HPV16 would suppress rather than enhance EMT in our experiments. However, the current results support previous findings suggesting that episomally replicating HPV16, from which all of the viral genes are expressed at normal levels, promotes differentiation to support its own differentiation-dependent life cycle [56]. EMT is known to suppress normal keratinocyte differentiation [83,84], so it is perhaps not surprising that HPV16 would seek to evade EMT. Of note, many but not all of the suppression of EMT-related factors by HPV16 were associated with the expression E5, an often-neglected HPV16 gene that we previously found to be responsible for promoting epithelial differentiation).

Taken together, this study provides novel evidence that stromal IRF3, a transcription factor widely known to be involved in innate immunity and host response, can also impact epithelial differentiation through the regulation of the EMT process, suggesting a possible crosstalk between innate immune response and wound healing. Furthermore, regulating the balance between EMT and differentiation has implications for the replication of HPV and perhaps other viruses that infect the epithelium. Future studies will be needed to further understand the signaling processes underlying this novel activity of IRF3 and its implications for antiviral immunity.

## Data Availability

Data are available at the National Center for Biotechnology Information Gene Expression Omnibus database (accession #GSE294286).

## Supplementary Materials

The following supporting information can be downloaded at https://www.mdpi.com/article/10.3390/v17050598/s1. Supplementary Table S1. Antibodies used in this study. Supplementary Table S2. Oligonucleotides used in this study. Supplementary Table S3. RNA-seq data—all genes. Supplementary Table S4. RNA-seq data—interferon-related genes.

## Abbreviations

ChIP	chromatin immunoprecipitation
EMT	epithelial-to-mesenchymal transition
FGF	fibroblast growth factor
GAPDH	glyceraldehyde 3-phosphate dehydrogenase
HBEGF	heparin-binding epidermal growth factor
HFF	human foreskin fibroblast
HFK	human foreskin keratinocyte
HPV	human papillomavirus
IFN	interferon
IL6	interleukin 6
IRF3	interferon regulatory factor 3
ISG	interferon-stimulated gene
KD	knockdown
NTC	non-target control
PIC	poly I
C (PIC)	polyinosinic-polycytidylic acid
RT-qPCR	reverse transcriptase–quantitative polymerase chain reaction
TGFβ	transforming growth factor beta
