# Supplementary material for: Stromal Interferon Regulatory Factor 3 Can Antagonize Human Papillomavirus Replication by Supporting Epithelial-to-Mesenchymal Transition
Source: Viruses. 2025 Apr 23;17(5):598. doi: 10.3390/v17050598 (PMC12115382; doi:10.3390/v17050598)
Supplement: Supplementary file 1 [file viruses-17-00598-s001.zip › Supplementary table S1-S2, S4.pdf]

Supplementary Table S1 Antibodies used in this study				
Name	Company	Catalog#	Dilution	Application
Beta Catenin	BD	610154	1:50	Immunofluorescence (IHC)
E-Cadherin	BD	610182	1:50	Immunofluorescence (IHC)
FILAGGRIN	Santa Cruz	Sc-66192	1:50	Immunofluorescence (IHC)
GAPDH	Santa Cruz	sc-47724	1:1000	Western Blotting
IRF3	Abcam	ab25950	1:1000	Immunofluorescence, ChIP
TGFβ1	Abcam	ab215715	1:1000	Western Blotting
ALEXA FLUOR 488 GOAT ANTI-MOUSE IgG	Life Tech	A11029	1:500	Immunofluorescence
ALEXA FLUOR 488 GOAT ANTI-MOUSE IgG	Life Tech	A11029	1:1000	Immunofluorescence

Supplementary Table S2. Oligonucleotides used in this study	
Target gene	Primer sequence
FLG	3’ TCCCCTACGCTTTCTTGTCCT<br>5’ TGAAGCCTATGACACCACTGA
HBEGF	3’ TTAGTCATGCCCAACTTCACTTT<br>5’ ATCGTGGGGCTTCTCATGTTT
HGF	3’ TCTTCAGTGCTGGATCTATTTTGATTAG<br>5’ TGCTGCAGCATGTCCTCCT
IFIT1	3’ TCTCAGAGGAGCCTGGCTAA<br>5’ TGCTCCAGACTATCCTTGACCT
IFNB	3’ TCATCCTGTCCTTGAGGCAGT<br>5’ CAGCAATTTTCAGTGTCAGAAGC
IL6	3’ GCAAGTCTCCTCATTGAATCC<br>5’ GGCACTGGCAGAAAACAACC
IRF3	3’ CCTGAGTTCACAAACTCGTAGAT<br>5’ GCAAAGAAGGGTTGCGTTTAG
FGF7	3’ TTGCTGTGACGCTGTTTGCTAT<br>5’ CAGCTGAGAAATAGTTTGTAGCTACAGTAGA
KRT6B	3’ ACTGCATCAGAAGGTACATCAC<br>5’ GCTAACCATCCTGCCCATTAT
KRT10	3’ CTACCTCATTCTCATACTTCAGCCTG<br>5’ AGCCTCGTGACTACAGCAAATACTAC
LOR	3’ AGAGGTCTTCACGCAGTCCACT<br>5’ CTCTCCTCACTCACCCTTCCTG
TGFB1	3’ CACGGGTTCAGGTACCGC

	5’ GAGGTCACCCGCGTGCTA
TGFB2	3’ CTGCACATCGTCCTGTGG<br>5’ GGAAACTTGACTGCACCGTT
TGFB3	3’ TTTCTCCACTGAGGACACATTGAA<br>5’ GCACCCAGGAAAACACCG
TGFBR2	3’ CTGCACATCGTCCTGTGG<br>5’ GGAAACTTGACTGCACCGTT
TGM1	3’ AGCTCGTCGTACTCATACTCGTCTG<br>5’ GCTGGAGATGGCACCATCC
SNAI1	3’ TCCCAGATGAGCATTGGCA<br>5’ CCCAATCGGAAGCCTAACTACAG
SNAI2	3’ GAGGATCTCTGGTTGTGGTATG<br>5’ AACTACAGCGAACTGGACAC
TWIST1	3’ GCTTGAGGGTCTGAATCTTGCT<br>5’ GTCCGCAGTCTTACGAGGAG
TWIST2	3’ CCCAAACATAAGACCCAGAAGA<br>5’ CAGTGACATCGGACAGAAGAC
VIM	3’ GGGTGTTTTCGGCTTCCTCT<br>5’ AGCTAACCAACGACAAAGCCC
ZEB1	3’ CGTTCTTCCGCTTCTCTCTTAC<br>5’ CTTCTCACACTCTGGGTCTTATTC
ZEB2	3’ AGCCTGAGAGGAGGATCACA<br>5’ CAACTCCGATGAACTGCTGA
TGFB1 Pro<br>IRF3 site 1	3’ GCAGCCTCCTGTCACTCAACAC<br>5’ CTAGGACCTCGGGGTCCCT
TGFB1 Pro<br>IRF3 site 2	3’ AGGGTCTGTCAACATGGGGG<br>5’ TCCTGACCCTTCCATCCTTCAGG
TGFB1 Pro<br>IRF3 site 3	3’ CCCCCATGTTGACAGACCCTC<br>5’ GGGGATGAGACACAGGGGAG

Supplementary Table S4

**TPMs**	**Stroma**			**Epithelium**	
Gene	NTC	IRF3 KD		NTC	IRF3 KD
IFNA1	0.00	0.00		0.00	0.00
IFNA10	0.00	0.00		0.00	0.00
IFNA10	0.00	0.00		0.00	0.00
IFNA11P	0.00	0.00		0.00	0.00
IFNA12P	0.00	0.00		0.00	0.00
IFNA13	0.00	0.00		0.00	0.00
IFNA14	0.00	0.00		0.00	0.00
IFNA16	0.00	0.00		0.00	0.00
IFNA17	0.00	0.00		0.00	0.00

IFNA2	0.00	0.00		0.00	0.00
IFNA20P	0.00	0.06		0.00	0.00
IFNA21	0.00	0.00		0.00	0.00
IFNA22P	0.05	0.00		0.00	0.00
IFNA4	0.00	0.00		0.00	0.00
IFNA5	0.00	0.00		0.00	0.00
IFNA6	0.00	0.00		0.00	0.00
IFNA7	0.00	0.00		0.00	0.00
IFNA8	0.00	0.00		0.00	0.00
IFNB1	0.00	0.04		0.00	0.00
IFNE	0.45	1.00		0.48	0.75
IFNG	0.00	0.00		0.03	0.00
IFNL1	0.00	0.00		0.03	0.07
IFNL2	0.00	0.00		0.00	0.02
IFNL3	0.00	0.05		0.04	0.00
IFNL4	0.02	0.03		0.02	0.14
IFNW1	0.00	0.00		0.00	0.00
